# Effect on inclined medial proximal tibial articulation for varus alignment in advanced knee osteoarthritis

**DOI:** 10.1186/s40634-019-0180-x

**Published:** 2019-03-28

**Authors:** Tomoharu Mochizuki, Yoshio Koga, Osamu Tanifuji, Takashi Sato, Satoshi Watanabe, Hiroshi Koga, Koichi Kobayashi, Go Omori, Naoto Endo

**Affiliations:** 1 Division of Orthopaedic Surgery, Department of Regenerative and Transplant Medicine, Niigata University Graduate School of Medical and Dental Science, 1-757 Asahimachi-dori Chuo-ku, Niigata, 951-8510 Japan; 2 Department of Orthopaedic Surgery, Nioji Onsen Hospital, Niigata, Japan; 3 Department of Orthopaedic Surgery, Niigata Medical Center, Niigata, Japan; 4 School of Health Sciences, Faculty of Medicine, Niigata University, Niigata, Japan; 5 Department of Health and Sports, Faculty of Health Sciences, Niigata University of Health and Welfare, Niigata, Japan

**Keywords:** Knee osteoarthritis, Weight-bearing conditions, Inclination in the medial compartment of the proximal tibia, Varus alignment, Tibial parallel phenomenon

## Abstract

**Background:**

The inclination of the medial compartment of the proximal tibia (MCT) is assumed to be a critical factor for varus alignment in advanced knee osteoarthritis (OA). This study was aimed at investigating; (1) whether the inclination of MCT is aligned parallel to the ground under weight-bearing (WB) conditions; (2) whether this is associated with the change in alignment and the relative position between the bones; and (3) whether the tibia or femur mainly contributes to the changes.

**Methods:**

We examined 102 knees (84 women, 18 men; mean 75 years). A three-dimensional (3D) assessment system was applied on biplanar whole lower extremity radiographies using 3D-to-2D image registration technique. The evaluation parameters were 1) MCT angle, 2) femorotibial angle (FTA), 3) medial-lateral femoral location to the tibia (M-L femoral location), 4) WB line passing point, and 5) tibial position to WB line (tibial position) and 6) femoral postion to WB line (femoral position). Each parameter was evaluated in non-WB and WB conditions, and the differences (Δ-parameters).

**Results:**

MCT angle in the world coordinate system was larger than that in the tibial coordinate system (*p* <  0.0001). ΔMCT angle was correlated with ΔFTA (*p* = 0.002) and ΔM-L femoral location (*p* = 0.004). The tibial position was the more dominant factor for ΔMCT angle (*p* = 0.001), ΔFTA (*p* <  0.0001), and ΔWB line passing point (*p* <  0.0001) .

**Conclusions:**

The inclination in MCT was aligned parallel to the ground under WB conditions (tibial parallel phenomenon). The parallel phenomenon was associated with the change of alignment and the relative position between the bones in the coronal plane. These phenomena were produced mainly by the tibia, not the femur.

**Level of evidence:**

Level IV.

## Background

Humans hold the body spinally erect in a multisegmental “antigravity pole” and support the body weight exclusively by vertical balance for bipedal locomotion (Skoyles, 2006). To realize stable bipedal locomotion, the shapes and motion of the load joints evolved. The distal articular surface in the loaded joint holding the proximal articular surface has the important role of aligning parallel to the ground for its antigravity action. For instance, at the knee joint, the tibia is crucial for holding the balance and realizing bipedalism (Mochizuki et al., 2018b).

The inclination of an articular surface in the medial compartment of the proximal tibia (MCT) is a critical factor for varus knee osteoarthritis (OA) (Cook et al., 1989; Matsumoto et al., 2015; Higano et al., 2016). The structure of the proximal tibial metaphysis is mechanically weak due to its low bone mineral density (BMD) and has laterality between the medial and lateral knee compartments OA (Lo et al., 2006; Akamatsu et al., 2009). The laterality of BMD can affect the proximal tibial inclination. Also, the tibial plateau inclination is different between healthy subjects and those with knee OA (Matsumoto et al., 2015). Varus inclination is present on the surface of the tibial joint in 88% of cases of varus knee OA (Cook et al., 1989). The larger inclination in the medial condylar plateau has been reported to exist before the onset of varus knee OA in advanced OA (Higano et al., 2016), and the steepening medial tibial plateau inclination may be the main contributor to the worsened varus deformity in knees with severe OA (Matsumoto et al., 2015).

**Fig. 1:**
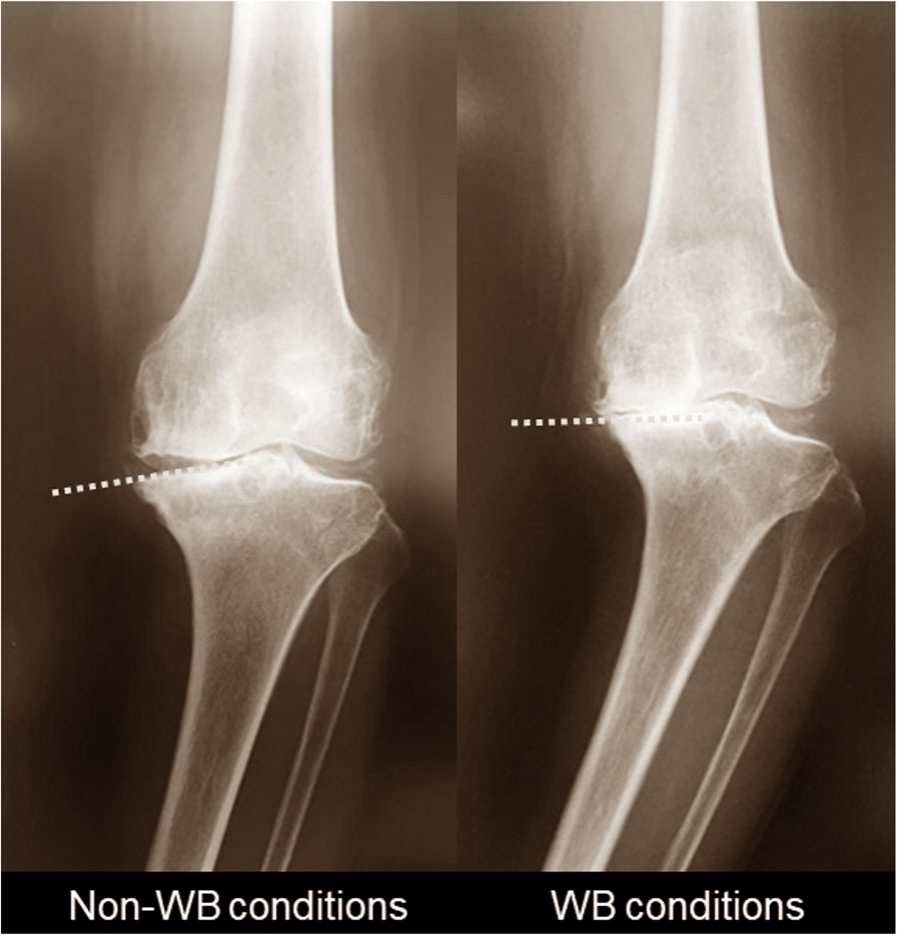
Knee radiographs in advanced knee OA under non-WB and WB conditions. An articular surface in the MCT under WB conditions was aligned parallel to the ground, compared to that under non-WB conditions

Varus malalignment of the lower extremity increases the risk of OA progression (Sharma et al., 2001). In addition to static malalignment under non-weight-bearing (WB) conditions, dynamic malalignment under WB conditions has been reported recently to be a major predictor for knee OA progression (Sharma et al., 2001; Chang A et al., 2004; Sharma et al., 2010). Coronal alignment under WB conditions theoretically produces the position change between the femur and tibia by slight motion in the standing position, yet the mechanism remains unknown. In the clinical setting, the general experience is that inclination of an articular surface in MCT is aligned more parallel to the ground under WB than non-WB conditions (parallel phenomenon) (Fig. 1). This phenomenon may be the key factor for varus malalignment leading to varus knee OA progression.

**Fig. 2:**
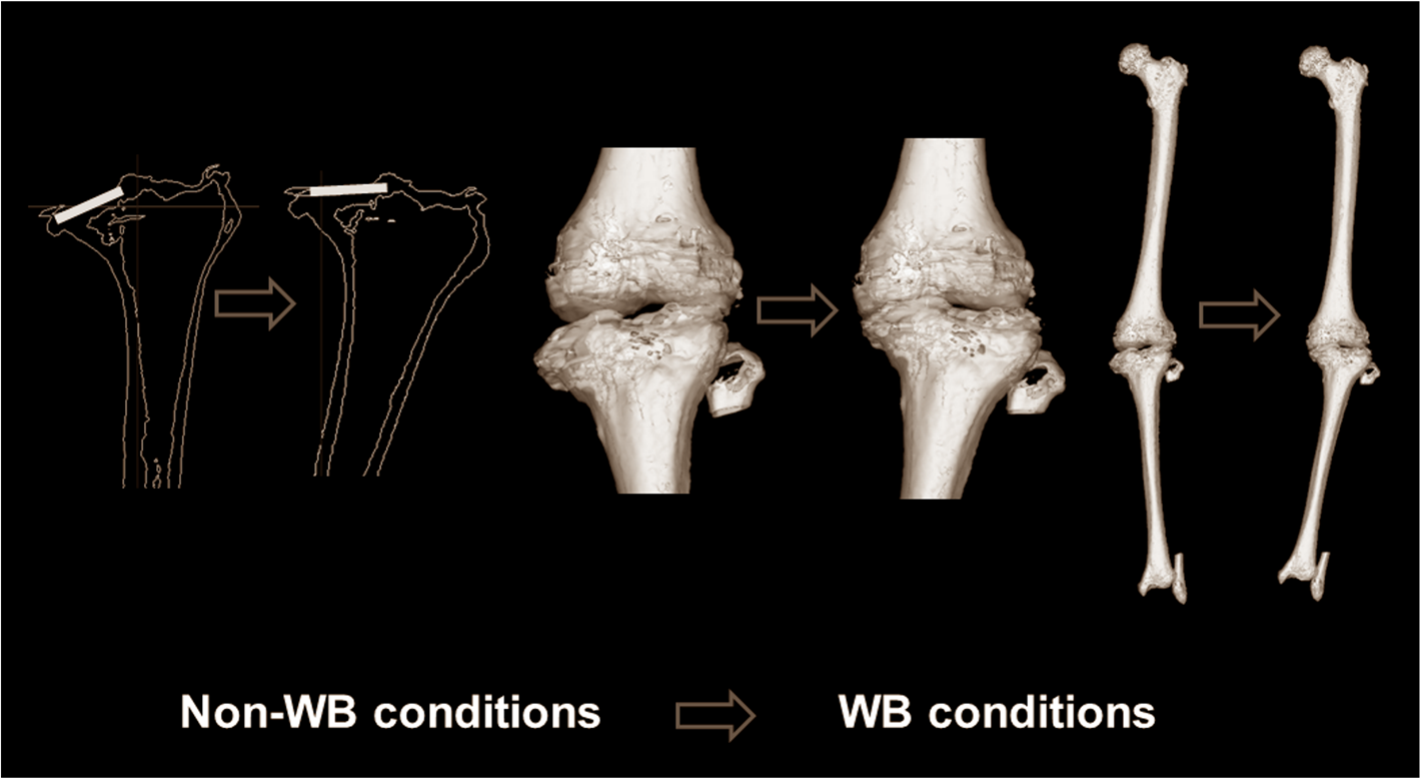
Schematic diagram showing the varus alignment mechanism in advanced knee OA. As the hypothesis, in a coronal plane, a tibial parallel phenomenon may produce varus malalignment and the position change between the femur and tibia under WB conditions in advanced knee OA

The main purpose of this study was to clarify that the parallel phenomenon in MCT is associated with the change in lower extremity alignment from non-WB to WB conditions. As the hypothesis (Fig. 2), we investigated whether: (1) an articular surface inclination in MCT tends to be aligned more parallel to the ground under WB conditions (parallel phenomenon), (2) this phenomenon is associated with the change in varus malalignment and the relative position between the femur and tibia, (3) the changes under WB conditions are larger in individuals with the WB line passing off an articular surface in MCT, and (4) they are associated with the changes in the tibia or femur.

## Methods

The institutional review board of Niigata University approved this study protocol (approval number, 2351). All subjects provided written or verbal informed consent to use the data. Among the 81 subjects, we examined 102 lower limbs (84 in 65 women, 18 in 16 men; mean age, 75 years; standard deviation, 6 years), which were classified as grades 3–4 based on the Kellgren–Lawrence classification by radiography images (Kellgren & Lawrence, 1957). Inclusion criteria were advanced varus knee OA indicating primary total or unicompartmental knee arthroplasty, high tibial osteotomy, and conservative treatment. Exclusion criteria were valgus OA, secondary OA after trauma, or other diseases.

**Fig. 3:**
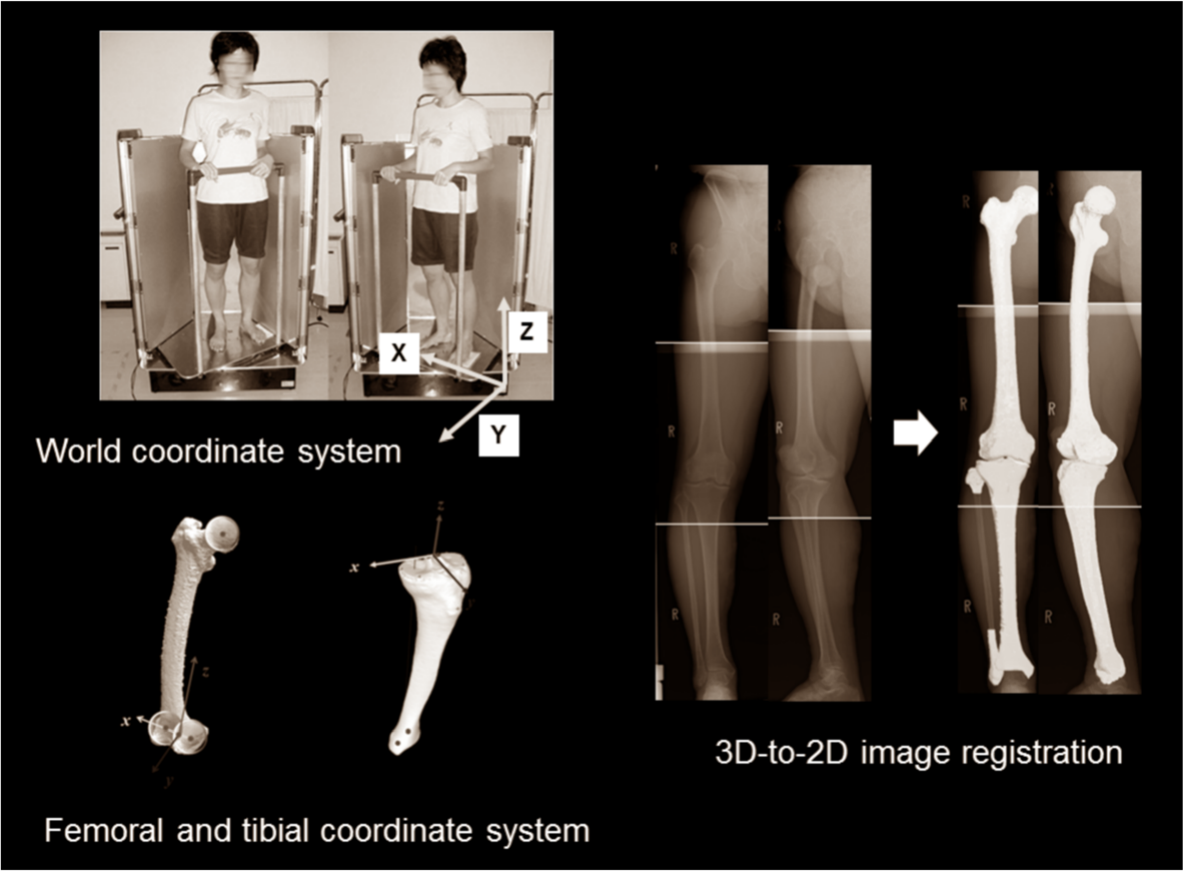
The 3D lower extremity alignment assessment system under WB conditions based on biplanar long lower extremity radiographs, applying the 3D-to-2D image registration techniques. 3D bone models and the anatomic coordinate system of the femur and tibia were reconstructed. The 3D bone model included the definitions of the parameters in the bony morphology and whole lower extremity alignment. To assess the 3D parameters “in the standing position” under WB conditions, biplanar long lower extremity radiographs were obtained in the standing position. Applying the 3D-to-2D image registration technique, projected outline points of each 3D model were the finite edge points of the 2D shadow created from the projections of all visible triangular surfaces of the 3D model. To precisely assess the position and angle to the ground, the world coordinate system was reconstructed

To test the hypotheses in a three-dimensional (3D) space, a special 3D assessment system for alignment and morphology under WB conditions was necessary. Our group developed the 3D lower extremity alignment assessment system under WB conditions (Knee CAS, LEXI, Inc., Tokyo, Japan) based on biplanar long lower extremity radiographs. This system used 3D-to-2D image registration techniques (Aiumi et al., 2010; Kobayashi et al., 2009; Mochizuki et al., 2013; Mochizuki et al.; 2014a, Mochizuki et al., 2014b; Mochizuki et al., 2015; Mochizuki et al., 2017a; Mochizuki et al., 2018a; Mochizuki et al., 2018b; Murayama et al., 2016; Sato et al., 2004; Takagi et al., 2017; Tanifuji et al., 2013; Watanabe et al., 2014). For the system overview, several major steps were necessary. First, the 3D bone model and anatomic coordinate system of the femur and tibia were constructed by computed tomography (CT) data (Fig. 3). Second, the definitions of the parameters in the bony morphology and whole lower extremity alignment were incorporated in the 3D bone models. At this time, bony parameters and the whole lower extremity alignment only “in the supine position” were calculated in the anatomic coordinate system because CT data were obtained in the supine position. To assess the 3D parameters “in the standing position” under WB conditions, one more step was necessary; thus, biplanar long lower extremity radiographs were obtained in the standing position. Appling 3D-to-2D image registration techniques, the information for the 3D bone model, including the parameters and the anatomic coordinate system, was incorporated in the biplanar long lower extremity radiographs. The 3D information “in the standing position” under WB conditions finally was acquired. To precisely assess the position and angle to the ground, the world coordinate system was reconstructed. The details in these steps are described below.

### 3D bone models and coordinate system

A 3D digital model of the femur and tibia was reconstructed from the CT images (SOMATOM Sensation 16; Siemens, Inc., Munich, Germany) using 3D visualization and modeling software (Zed View; LEXI, Inc., Tokyo, Japan). The anatomic tibial and femoral coordinate systems were established by referencing several bony landmarks, as defined previously (Sato et al., 2004) (Fig. 3). For the femoral coordinate system, the geometric center axis (a line connecting the centers of spheres representing the medial and lateral posterior femoral condyles) was defined as the femoral *x*-axis (positive right). The origin of the coordinate system was the midpoint between the centers of these posterior condylar spheres. The femoral *y*-axis (positive anteriorly) was a perpendicular line to the plane formed by the femoral head center and two centers of the posterior condylar spheres. The femoral *z*-axis (positive superiorly) was the cross-product of the *x*- and *y*-axes. For the tibia, the *z*-axis was defined by a line connecting the midpoint of the tibial eminences and those of the medial and lateral tops of the talar dome (positive superiorly). The tibial *y*-axis (positive anteriorly) was the line perpendicular to the *z*-axis from the mediolateral center of the tibial insertion of the posterior cruciate ligament. The tibial *x*-axis was the cross-product of the *z*- and *y*-axes (positive right). To detect the position of the subjects to the ground, the world coordinate system was constructed, defined in the acrylic box for the calibration (*z*-axis, positive superiorly; *y*-axis, positive anteriorly; *x*-axis, positive right). Precisely, the origin point was set in the position of the photography platform as shown in Fig. 3. Based on the world coordinate system, the angle and position to the ground of each parameter were determined.

### 3D-to-2D image registration technique in Biplanar radiographs

Biplanar radiographs of the entire lower extremity were obtained under WB conditions in the standing position with the knee fully extended and toes neutral. A biplanar radiography system was applied to capture frontal and oblique x-ray images. The rotation table was set at 0° and 60° relative to the optical axis of the x-ray source. For each table position, the x-ray tube was calibrated beforehand to determine the projection matrix (Faugeras, 1993), which provided 3D positioning of the focus of the x-ray source. Contours of the femur and tibia in biplanar radiographs were detected according to the method described by Canny (1986). Projected outline points of each 3D model were the finite edge points of the 2D shadow produced from the projections of all visible triangular surfaces of the 3D model (Kobayashi et al., 2009). Then, this 3D-to-2D image registration technique enabled 3D digital bone models to be projected onto biplanar radiographs (Ariumi et al., 2010; Kobayashi et al., 2009; Mochizuki et al., 2017a; Sato et al., 2004) (Fig. 3). After the image-matching procedures, a 3D view of the digital bone model that accurately reproduced the spatial relationship between the femur and tibia at a projection in biplanar radiographs was displayed, and all alignment and bony parameters were calculated automatically.

The accuracy of the 3D-to-2D image registration technique was established as follows: three spherical markers were attached to each sawbone of the femur and tibia to determine the local coordinate system. Outlines of the 3D bone models were projected on extracted contours of each femur and tibia in the frontal and oblique radiographs. The 3D position of each model was recovered by minimizing the difference between the projected outline and contour. Median and maximum values of the absolute error in estimating the relative positions for the femur to tibia were within 0.5 mm and 0.6°, and 1.6 mm and 1.5°, respectively (Kobayashi et al., 2009).

### Evaluation parameters in coronal Planes of each coordinate system

**Fig. 4:**
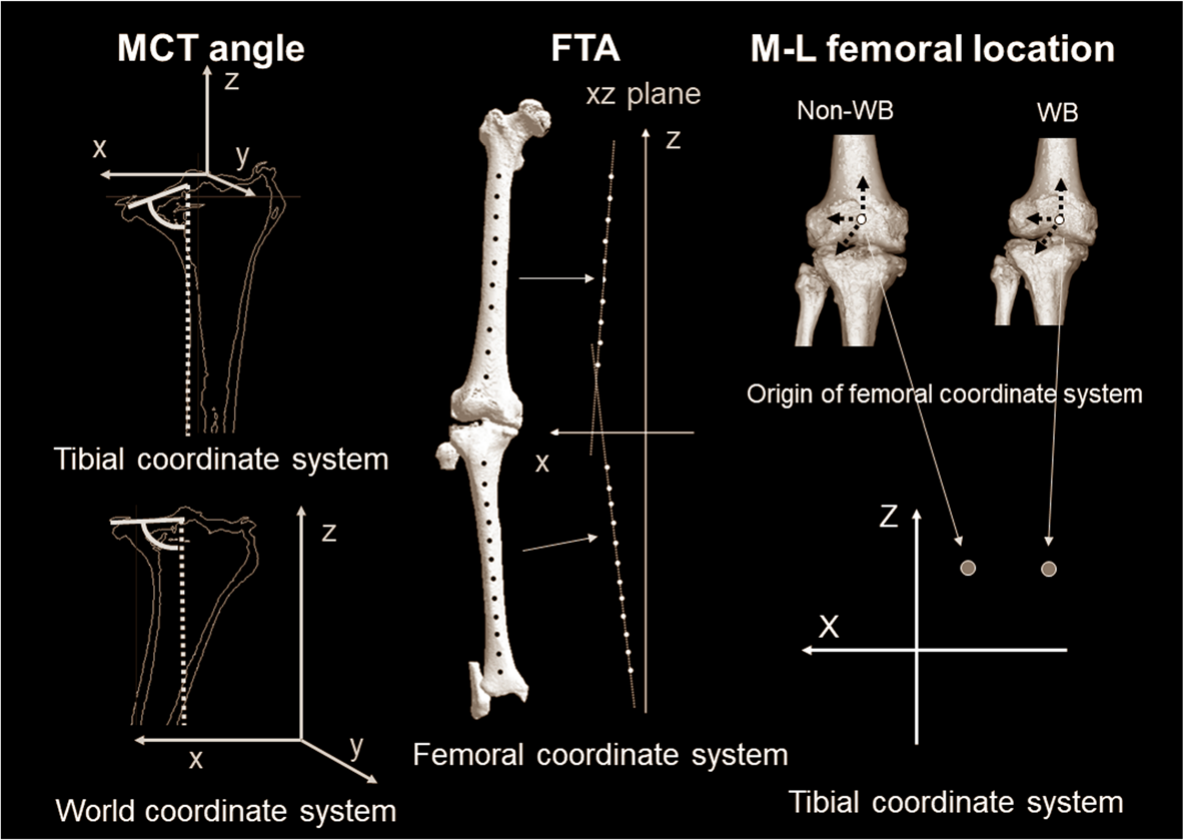
Schematic diagram showing the MCT angle, FTA, and M–L femoral location. MCT angle under non-WB conditions in the tibial coordinate system was defined as the angle between the tangential line of an articular surface in MCT and the *z*-axis of the tibial coordinate system. As the assessment plane, the coronal plane passing the tibial coordinate system origin point was used. MCT angle under WB conditions in the world coordinate system was defined as the angle between the tangential line of an articular surface in the MCT and the *z*-axis of the world coordinate system. As the assessment plane, the coronal plane passing the middle section between the anterior- and posterior-most points of the medial compartment in the world coordinate system was used. In terms of FTA, the anatomic longitudinal axes were defined as a regression line obtained from approximating distances from these 10 centroids in the femur and 12 centroids in the tibia by the least squares method, respectively. FTA was assessed as the angle between the anatomic longitudinal axes of the femur and tibia in the coronal plane of the femoral coordinate system. Regarding M–L femoral location, the M–L location of the femur relative to the tibia was defined as the location of the origin point of the femoral coordinate system, and was assessed in the coronal plane of the tibial coordinate system

**Fig. 5:**
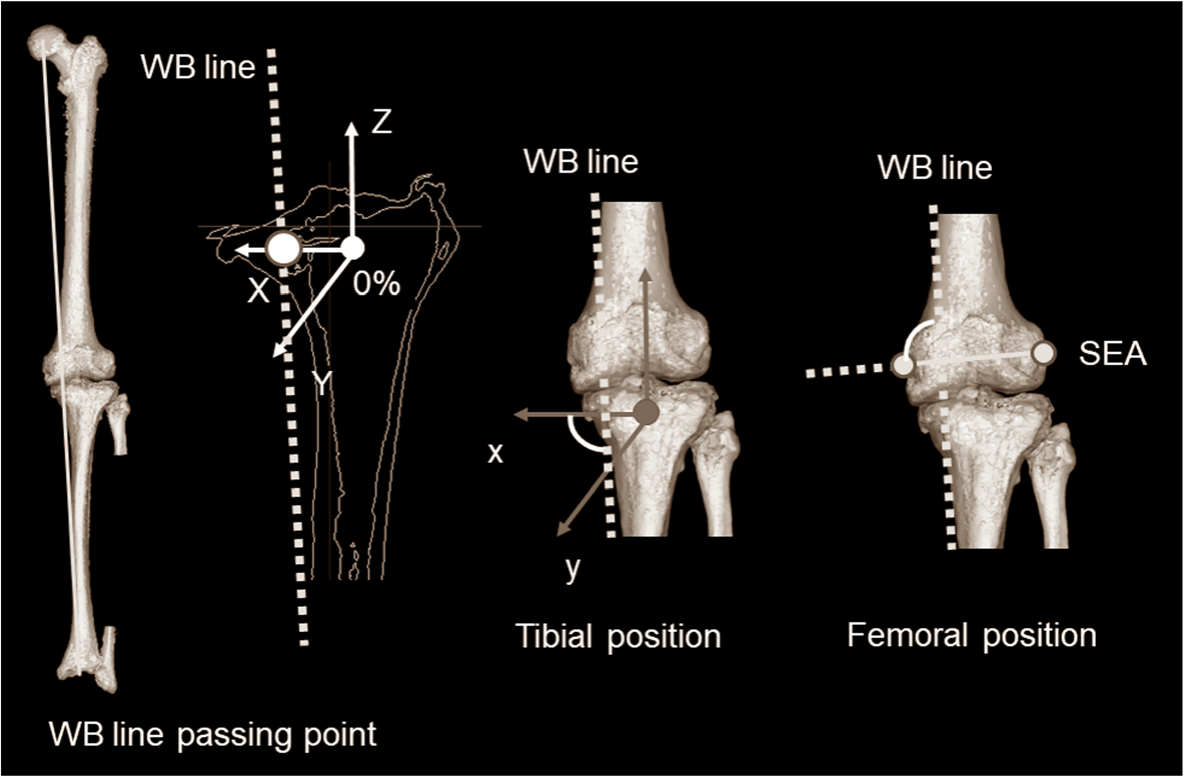
Schematic diagram showing the WB line passing point, tibial position, and femoral position. Regarding WB line passing point, WB line was defined as the line connecting the femoral head with the center of the ankle joint in a 3D space. WB line passing point was the location of the WB line on the *xy* plane in the tibial coordinate system. The WB line in the tibial coordinate system was described by percent indication (0% = origin in the tibial coordinate system, + 100% = medial-most point in MCT, and ≥ 100% = passing point outside from MCT). The tibial position was defined as the angle between the WB line and the *x*-axis in the tibial coordinate system. The femoral position was defined as the angle between the WB line and the surgical epicondylar axis in the tibial coordinate system

The evaluation parameters were as follows (Figs. 4 and 5): (1) MCT angle, (2) femorotibial angle (FTA), (3) medial–lateral (M–L) femoral location – the M–L location of the femur relative the tibia, (4) WB line passing point – the passing point of the WB line through an articular surface in MCT, (5) tibial position – the relative angle between the WB line and *x*-axis of the tibial coordinate system, and (6) femoral position – the relative angle between the WB line and surgical epicondylar axis of the femur. In each parameter, the difference between non-WB and WB conditions was presented (Δ-parameter).

MCT angle under non-WB conditions in the tibial coordinate system showed the inclination of an articular surface in MCT as the morphology and was defined as the angle between the tangential line of an articular surface in MCT and the *z*-axis of the tibial coordinate system. As the assessment plane, the coronal plane (*xz*) passing the origin point in the tibial coordinate system was used (Fig. 4). A lower MCT angle in the tibial coordinate system indicated a larger inclination of an articular surface in MCT. MCT angle under WB conditions in the world coordinate system showed the inclination of an articular surface in MCT to the ground under WB conditions and was defined as the angle between the tangential line of an articular surface in MCT and the *z*-axis of the world coordinate system. As the assessment plane, the coronal plane (*xz*) passing the middle section between the anterior- and posterior-most points of the medial compartment in the world coordinate system was used (Fig. 4). A larger MCT angle in the world coordinate system indicated that the inclination of an articular surface in MCT is more parallel to the ground under WB conditions. The difference in MCT angle between non-WB conditions in the tibial coordinate system and WB conditions in the world coordinate system was defined as the Δ-parameter.

FTA was defined in a 3D space, according to a previously published method (Ariumi et al., 2010) (Fig. 4). A point group centroid was calculated automatically for the 10 respective cross-sectional planes of the femoral diaphysis in the femoral coordinate system and 12 respective cross-sectional planes of the tibial diaphysis in the tibial coordinate system. The anatomic longitudinal axes were defined as a regression line obtained from approximating distances from these 10 centroids in the femur and 12 centroids in the tibia by the least squares method, respectively. FTA was assessed as the angle between the anatomic longitudinal axes of the femur and tibia in the coronal plane (*xz*) of the femoral coordinate system under non-WB and WB conditions. The differences in FTA in the femoral coordinate system between non-WB and WB conditions were defined as the Δ-parameters of FTA.

M–L femoral location relative to the tibia was defined as the location of the origin point of the femoral coordinate system and was assessed in the coronal plane (*xz*) of the tibial coordinate system under non-WB and WB conditions (Fig. 4). The plus or minus values for the right or left side in the coordinate system were corrected (medial location, positive). The differences in M–L femoral location in the tibial coordinate system between non-WB and WB conditions were defined as the Δ-parameters.

In terms of the WB line passing point, the WB line was defined as the line connecting the femoral head with the center of the ankle joint in a 3D space. WB line passing point was the location of the WB line on the *xy* plane in the tibial coordinate system. The WB line was described by percent indication (0% = the origin in the tibial coordinate system, + 100% = the medial-most point in MCT, and ≥ 100% = the passing point outside from MCT) (Fig. 5). The Δ-parameter was the difference in WB line passing point in the tibial coordinate system between non-WB and WB conditions.

To identify whether the change in lower extremity alignment by WB conditions was associated with movement in the tibia or femur, the position changes of the femur and tibia from non-WB to WB conditions were investigated in the coronal plane (*xz*) of the tibial coordinate system. As it was assumed that the femur or tibia moved relative to the WB line by WB conditions, the association between the WB line and the parameter of each bone was investigated. In the tibia, the angle between the WB line and the *x*–axis in the tibial coordinate system was defined as the tibial position (Fig. 5). In the femur, the angle between the WB line and the surgical epicondylar axis in the tibial coordinate system was defined as the femoral position (Fig. 5). The differences in tibial and femoral positions in the tibial coordinate system between non-WB and WB conditions were defined as the Δ-parameters, respectively.

### Statistical analysis

The parameters categorized between non-WB and WB conditions were compared with a paired *t*-test when the data had a normal distribution and equal variance, and the Wilcoxon signed-rank test when the data had no normal distribution. To compare the parameters categorized in 100% of the WB line, the (1) two sample *t*-test, (2) Welch test, and (3) Mann–Whitney *U* test were applied when the data had (1) a normal distribution and equal variance, (2) a normal distribution but no equal variance, and (3) no normal distribution, respectively. The correlation between each parameter was evaluated using Pearson’s product moment and Spearman’s rank-order correlations when the data did and did not have a normal distribution, respectively. Multiple linear regression analysis was applied to identify the contribution of each bone (femur or tibia) to the change from non-WB to WB conditions. *P* <  0.01 indicated statistical significance (Version 21; SPSS, Inc., Chicago, IL, USA). When comparing the groups under non-WB and WB conditions, the post hoc statistical power analysis showed: MCT angle: power = 1.000, *p* <  0.0001; FTA: power = 0.965, *p* <  0.0001; M–L femoral location: power = 0.982, *p* <  0.0001; WB line passing point: power = 0.950, *p* <  0.0001; tibial position: power = 0.993, *p* <  0.0001; femoral position: power = 0.226, *p* = 0.227.)

## Results

All parameters showed significant differences between non-WB and WB conditions (Table 1). MCT angle was larger under WB than non-WB conditions, which meant that an articular surface in MCT was aligned parallel to the ground under WB conditions (*p* <  0.0001). FTA showed more varus alignment under WB conditions (*p* <  0.0001). M–L femoral location demonstrated that the femur more medially located to the tibia under WB conditions (*p* <  0.0001). WB line passing point was more medial under WB conditions (*p* <  0.0001). Tibial (*p* <  0.0001) and femoral position (*p* <  0.0001) to the WB line changed from non-WB to WB conditions.

**Table 1:** Evaluation parameters

	Non-WB condition		WB condition		Δ parameters		Comparison between non-WB and WB conditions
	Mean	95% CI	Mean	95% CI	Mean	95% CI	*p* value
MCT angle (°)	78.3	76.9–79.7	87.6	86.6–88.5	9.3	8.0–10.5	< 0.0001*
FTA (°)	185.4	184.5–186.3	188.1	187.0–189.2	2.7	2.1–3.3	< 0.0001*
M-L femoral location (mm)	5.7	5.0–6.4	8.0	7.1–8.8	2.3	1.5–3.0	< 0.0001*
WB line passing point (%)	99.4	90.6–108.2	124.6	114.0–135.2	25.2	20.6–29.8	< 0.0001*
Tibial position (°)	96.0	95.5–96.6	98.0	97.3–98.7	2.0	1.6–2.4	< 0.0001*
Femoral position (°)	93.7	93.0–94.4	94.3	93.6–95.0	0.6	0.3–0.9	< 0.0001*

*MCT* = medial compartment in the proximal tibia, *WB* = weight-bearing, *Δ difference* = difference in each parameter between non-WB and WB conditions, *tibial position* = the relative angle of WB line to *x*-axis in the tibial coordinate system, *femoral position* = the relative angle of WB line to a surgical epicondylar axis, 95%CI = 95% confidence interval; *Significant difference = *p* <  0.01

Regarding the correlation (Table 2), ΔMCT angle was correlated with ΔFTA (correlation coefficient [CC] = 0.305, *p* = 0.002), ΔM–L femoral location (CC = 0.283, *p* = 0.004), and ΔWB line passing point (CC = 0.359, *p* <  0.0001), indicating that the parallel phenomenon in MCT was associated with more varus alignment, more medial location of the femur to the tibia, and more medial WB line passing point from non-WB to WB conditions. MCT angle in the tibial coordinate system was highly correlated with ΔMCT angle (CC = − 0.729, *p* <  0.0001), which indicated that the steeper inclination in MCT was associated with a larger parallel phenomenon in MCT. ΔTibial position was correlated with ΔMCT (CC = 0.338, *p* = 0.001), ΔFTA (CC = 0.557, *p* <  0.0001), and ΔWB line passing point (CC = 0.699, *p* <  0.0001), while Δfemoral position was not correlated with all parameters. This result suggested that the change in tibial, not femoral, position was associated with the parallel phenomenon in MCT, more varus alignment, and more medial WB line passing point under WB conditions.

**Table 2:** Correlation between each parameter

	MCT angle tibial coordinate system		ΔMCT angle		ΔTibial position		ΔFemoral position	
	CC	*p* value	CC	*p* value	CC	*p* value	CC	*p* value
ΔMCT angle	−0.729	< 0.0001*	–	–	0.338	0.001*	0.158	0.113
ΔFTA	−0.165	0.098	0.305	0.002*	0.557	< 0.0001*	0.145	0.145
ΔM-L femoral location	−0.286	0.004*	0.283	0.004*	0.179	0.071	−0.053	0.600
ΔWB line passing point	−0.234	0.009*	0.359	< 0.0001*	0.699	< 0.0001*	0.122	0.221
ΔTibial position	−0.259	0.009*	0.338	< 0.0001*	–	–	−0.004	0.966
ΔFemoral position	−0.023	0.820	0.158	0.113	−0.004	0.966	–	–

*CC* = correlation coefficient, *MCT* = medial compartment in the proximal tibia, *WB* = weight-bearing, *Δ difference* = difference in each parameter between non-WB and WB conditions, *tibial position =* the relative angle of WB line to *x*–axis in the tibial coordinate system, *femoral position* = the relative angle of WB line to a surgical epicondylar axis, *95%CI* = 95% confidence interval; *Significant difference = *p* < 0.01

The data in the groups with 100% of WB passing point under WB conditions are shown in Table 3. The changes in most parameters under WB conditions became larger when the WB line passed off the articular surface of MCT.

**Table 3:** Evaluation parameters of the groups categorized with 100% of WB passing point in WB condition

	< 100% of WB passing point (*n* = 36)		≥100% of WB passing point (*n* = 66)		
	Mean	95% CI	Mean	95% CI	*p* value
ΔMCT angle (°)	4.7	3.1–6.2	11.8	10.3–13.3	< 0.0001*
ΔFTA (°)	1.5	0.6–2.5	3.3	2.7–4.0	0.002*
ΔM-L femoral location (mm)	0.9	0.1–1.7	3.0	2.0–4.1	< 0.0001*
ΔWB line passing point (%)	12.6	5.2–20.1	32.1	26.7–37.4	< 0.0001*
ΔTibial position (°)	1.1	0.6–1.6	2.5	2.0–3.0	< 0.0001*
ΔFemoral position (°)	0.5	0.0–1.0	0.7	0.3–1.0	0.661

*MCT* = medial compartment in the proximal tibia, *WB* = weight-bearing, *Δ difference* = difference in each parameter between non-WB and WB conditions, *tibial position* = the relative angle of WB line to *x*–axis in the tibial coordinate system, *femoral position* = the relative angle of WB line to a surgical epicondylar axis, *95%CI* = 95% confidence interval; *Significant difference = *p* < 0.01

In Table 4, multiple linear regression analysis revealed that Δtibial position was the more dominant factor for ΔMCT angle (*p* = 0.001), ΔFTA (*p* <  0.0001), and ΔWB line passing point (*p* <  0.0001) than Δfemoral position (Table 4).

**Table 4:** Multiple linear regression analysis

Dependent variable	Independent variable	Beta	*t* value	*p* value
ΔMCT angle	ΔTibial position	0.338	3.587	0.001*
	ΔFemoral position	0.159	1.708	0.091
ΔFTA	ΔTibial position	0557	6.708	< 0.0001*
	ΔFemoral position	0.148	1.800	0.075
Δ M-L femoral location	ΔTibial position	0.106	1.060	0.292
	ΔFemoral position	−0.067	−0.670	0.504
ΔWB line passing point	ΔTibial position	0.701	9.833	< 0.0001*
	ΔFemoral position	0.097	1.360	0.177

*MCT* = medial compartment in the proximal tibia, *WB* = weight-bearing, *Δ difference* = difference in each parameter between non-WB and WB conditions, *tibial position* = the relative angle of weight-bearing line to *x*–axis in the tibial coordinate system, *femoral position* = the relative angle of weight-bearing line to a surgical epicondylar axis, *95%CI* = 95% confidence interval, *Significant difference = *p* < 0.01

## Discussion

The main finding of this study was that, in the coronal plane, MCT was aligned parallel to the ground under WB conditions (parallel phenomenon), and the parallel phenomenon in MCT was correlated with the change in varus alignment from non-WB to WB conditions for advanced varus knee OA.

The current study showed the tibial parallel phenomenon that MCT was aligned parallel to the ground under WB conditions for advanced varus knee OA (hypothesis 1). The mechanism of varus malalignment for advanced knee OA generally is explained by several factors, such as knee adduction moment (Foroughi, et al. 2009), looseness of the lateral collateral ligament (LCL), and degenerative cartilage damage (Koga, 2008). Varus malalignment in the lower extremity is associated with knee adduction moment (Moyer et al., 2010). Even in the tertile with the lowest mass, a 1.7 nm increase in peak knee adduction moment was confirmed for every 1° increase toward varus alignment (Moyer et al., 2010). With regard to medial-lateral laxity, even in normal knees, lateral and medial ligamentous laxities were not balanced and more lateral than medial ligamentous laxity has been observed (Okazaki et al., 2006). Understandably, in knee OA, increased varus–valgus laxity has also been reported (Ishii et al., 2009), and this increased laxity may influence disease progression and overall function (Freisinger et al., 2017). Regarding cartilage, for every 1° increase in varus angulation for coronal alignment, there was an annual loss of medial femoral cartilage in the longitudinal study (Cicuttini et al., 2004). Our study suggested that the biomechanical mechanism, such as knee adduction moment, malfunction of ligaments, and cartilage around a knee, may contribute to the parallel phenomenon of MCT under WB conditions in advanced knee OA. In detail, it is presumed that: (1) the degenerative cartilage does not efficiently absorb load bearing and (2) malfunction of the LCL and cruciate ligaments cannot repress large knee adduction moment, which assumingly causes a tibial parallel phenomenon in MCT for advanced knee OA. The association among these factors must be solved in the future.

The parallel phenomenon in MCT was associated with the change in lower extremity alignment and the relative position between the femur and tibia under WB conditions (hypothesis 2). With increasing varus malalignment, the moment arm for ground reaction force vector is increased, resulting in a higher adduction moment than that observed in the neutral knee (Schipplein & Andriacchi 1991). Knee adduction moment acts to force the tibia into varus (Birmingham et al., 2007; Zhao D et al., 2007), which means the tibial parallel phenomenon found in this study. The steeper inclination in MCT also showed the larger parallel phenomenon (Table 2). In terms of postual control, joint movements in the frontal plane are more unstable during single-leg standing, appearing as greater movements (Wang et al., 2014). The proportion of individuals using a postural control strategy that primarily uses the hip joint is reportedly greater than that using the ankle joint strategy (Aberg et al., 2011). A greater internal hip abduction moment during gait was associated with a reduced likelihood of medial tibiofemoral OA progression (Chang et al., 2005). Thus, besides knee adduction moment, hip abduction moment functions in advanced knee OA. The present study exhibited that, the tibia, but not the femur, mainly contributed to the changes of the parameters under WB conditions (hypothesis: 4). This fact may suggest that the main responsible factor of varus malalignment under WB conditions is the tibial parallel phenomenon due to knee adduction moment, while femoral abduction due to hip abduction moment is the slight motion, aligning the weight-bearing line perpendicular to the load-bearing surface of the tibia (MCT).

Sharma, et al. (2017) noted that varus thrust visualized during gait is associated with knee OA progression and should be a target of intervention development, but the accurate causes and mechanism remain unclear. The varus thrust is expressed as the momentary sideways movement of the knee (Chang et al., 2004; Jackson et al., 2004). In theory, the mechanism of varus thrust can be presumed by analyzing the changes in the alignment and the relative position of the bones between non-WB and WB conditions (Ogata et al., 1995). Our study showed that the parallel phenomenon in MCT was associated with the change in lower extremity alignment and the relative position between the bones under WB conditions (hypothesis 2), and the changes were drastic when the WB line passed off the articular surface of the MCT (hypothesis 3). As one of several possibilities, the parallel phenomenon in MCT may be a cause for varus thrust. It is assumed that, once the WB line passes off the MCT, as the knee adduction moment becomes larger, the parallel phenomenon of the MCT may be larger, leading to varus thrust.

Our study had several limitations. First, the sample size of men was relatively small, as varus knee OA is more common in females in Japan. Second, since this evaluation did not consider a torsional factor (Mochizuki et al., 2017b), MCT angle in this study strictly consisted of not only a coronal factor but also, more or less, a sagittal factor (posterior inclination of the tibia). If the most accurate assessment was required, the normal vector of the 3D plane in MCT should be applied. However, regardless of the inclusion of the sagittal factor, the MCT in our study was aligned more parallel under WB conditions. Considered from a different viewpoint, this fact unintentionally proved that the 3D MCT plane was aligned more parallel to the ground under WB conditions because the MCT angle included the coronal and sagittal factors. In other words, the coronal and sagittal planes might be aligned more parallel to the ground under WB conditions, but this evidence will be proved by further studies applying the normal vector of the 3D plane in MCT.

## Conclusions

The MCT inclination was aligned more parallel to the ground under WB conditions and was associated with the change in alignment and the relative position between the bones in the coronal plane. Once the WB line passed off the medial tibial plateau, the changes due to the parallel phenomenon were large. These phenomena were produced mainly by the tibia, not the femur.

## Abbreviations

2D: Two-dimensional
3D: Three-dimensional
MCT: Medial compartment of the proximal tibia
OA: Knee osteoarthritis
WB: Weight-bearing
LCL: Lateral collateral ligament (LCL)
